# Comparison of prognostic value between CAD-RADS 1.0 and CAD-RADS 2.0 evaluated by convolutional neural networks based CCTA

**DOI:** 10.1016/j.heliyon.2023.e15988

**Published:** 2023-05-04

**Authors:** Zengfa Huang, Yang Yang, Zheng Wang, Yunting Hu, Beibei Cao, Mei Li, Xinyu Du, Xi Wang, Zuoqin Li, Wanpeng Wang, Yi Ding, Jianwei Xiao, Yun Hu, Xiang Wang

**Affiliations:** a Department of Radiology, The Central Hospital of Wuhan, Tongji Medical College, Huazhong University of Science and Technology, Wuhan, Hubei, 430014, China; b Department of Community Health, Hanyang District Center For Disease Control and Prevention, Wuhan, Hubei, 430050, China

**Keywords:** Prognosis, CAD-RADS, Coronary artery disease, Coronary computed tomography angiography

## Abstract

**Objectives:**

The aim of the present study was to investigate the prognostic value of the novel coronary artery disease reporting and data system (CAD-RADS) 2.0 compared with CAD-RADS 1.0 in patients with suspectedcoronary artery disease (CAD) evaluated by convolutional neural networks (CNN) based coronary computed tomography angiography (CCTA).

**Methods:**

A total of 1796 consecutive inpatients with suspected CAD were evaluated by CCTA for CAD-RADS 1.0 and CAD-RADS 2.0 classifications. Kaplan-Meier and multivariate Cox models were used to estimate major adverse cardiovascular events (MACE) inclusive of all-cause mortality or myocardial infarction (MI). The C-statistic was used to assess the discriminatory ability of the two classifications.

**Results:**

In total, 94 (5.2%) MACE occurred over the median follow-up of 45.25 months (interquartile range 43.53–46.63 months). The annualized MACE rate was 0.014 (*95% CI*: 0.011–0.017). Kaplan-Meier survival curves indicated that the CAD-RADS classification, segment involvement score (SIS) grade, and Computed Tomography Fractional Flow Reserve (CT-FFR) classification were all significantly associated with the increase in the cumulative MACE (all *P* < 0.001). CAD-RADS classification, SIS grade, and CT-FFR classification were significantly associated with endpoint in univariate and multivariate Cox analysis. CAD-RADS 2.0 showed a further incremental increase in the prognostic value in predicting MACE (c-statistic 0.702, *95% CI*: 0.641–0.763, *P* = 0.047), compared with CAD-RADS 1.0.

**Conclusions:**

The novel CAD-RADS 2.0 evaluated by CNN-based CCTA showed higher prognostic value of MACE than CAD-RADS 1.0 in patients with suspected CAD.

## 1 Introduction

Coronary computed tomography angiography (CCTA) has gained clinical validation in the last decade and large-scale clinical trials have demonstrated its prognostic value for predicting major adverse cardiovascular events (MACE) and mortality [[1], [2], [3], [4]]. CCTA has been included in several guidelines. Some professional societies such as the European Society of Cardiology (ESC), and the UK National Institute for Health and Clinical Excellence (NICE) have recommended CCTA as the first-line test in patients with suspected coronary artery disease (CAD) [5,6].

In 2016, multiple societies in the radiology and cardiology communities introduced the Coronary Artery Disease Reporting and Data System (CAD-RADS) as a collaborative effort [7,8]. A higher CAD-RADS classification has been confirmed to be associated with increased risks of mortality and MACE [[9], [10], [11], [12]]. However, this classification was based only on stenosis degree and recent research has revealed that traditional CAD classification or Duke CAD prognostic index showed similar values for prediction of all-cause mortality or myocardial infarction (MI) [13,14]. Moreover, accumulated studies have demonstrated that assessment of plaque burden and functional testing enhanced the risk stratification in patients with stable chest pain or suspected CAD [[15], [16], [17], [18], [19]]. The recent updated 2022 CAD-RADS 2.0 classification followed a framework of stenosis, plaque burden, modifiers and ischemia evaluation which is intended to enhance patient management decisions based on CCTA [7]. However, the prognostic value of this new classification is unknown.

We have previously developed a deep convolutional neural network (CNN) based CAD-RADS for standardized reporting and achieved good consistency with radiologists in patients with suspected CAD [20]. In this study, we further aimed to investigate the prognostic value of the novel CAD-RADS 2.0 in patients with suspected CAD evaluated by CNN-based CCTA compared with CAD-RADS 1.0.

## 2 Methods

This was a retrospective, observational, single center study. The trial protocol was reviewed and approved by the ethics committee of the Central Hospital of Wuhan, Tongji Medical College, Huazhong University of Science and Technology and was conducted in compliance with the Health Insurance Portability and Accountability Act (HIPAA) of 1996. Written informed consent was waived because of its retrospective observational nature.

### 2.1 Study population

**Fig. 1:**
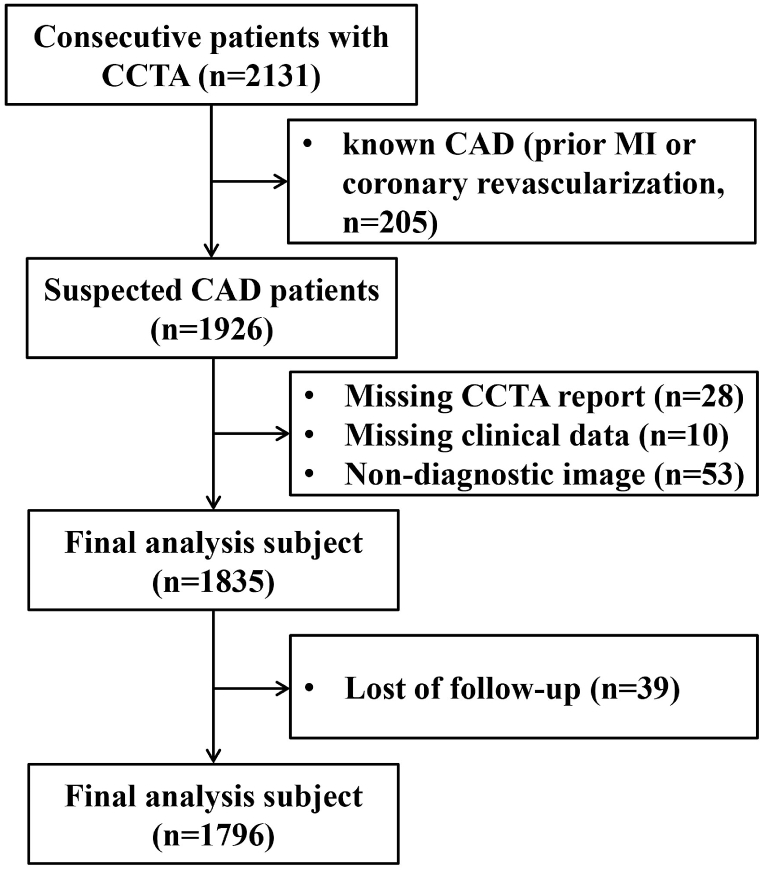
Flowchart of participant selection for analysis in the present study. CAD = coronary artery disease; CCTA = coronary computed tomography angiography; MI = myocardial infraction.

The study population consisted of 2131 consecutive inpatients with stable chest pain (suspected CAD) who were examined using CCTA between November 2018 and April 2019. Inclusion criteria were adults aged ≥18 years old, CCTA examination with a ≥64- detector row scanner and well-documented electronic records. Exclusion criteria were prior MI or coronary revascularization (n = 205), missing data, including CCTA report (n = 28) and clinical data (n = 10), non-diagnostic image (n = 53) or loss to follow-up (n = 39). Finally, a total of 1796 patients were included in the present study (Fig. 1).

### 2.2 CCTA protocol

CCTA was performed with a dual-source CT scanner (Somatom Definition, Siemens Medical Solutions, Forchheim, Germany). Beta-blockers were administered to lower the heart rate when a patients’ heart rate was greater than 65 beats/min before the scan. The prospectively or retrospectively ECG-triggered acquisition was used for the CCTA protocol. The scan parameters were as follows: detector collimation 128 × 0.6 mm; tube voltage 100 or 120 kV; mean tube current 280 mAs and temporal resolution 75 ms. Details of the CCTA protocol were as reported in our previous studies [[20], [21], [22]].

### 2.3 CAD-RADS analysis

All the CCTA images were automatically sent or manually uploaded to an AI ML platform (Computer Aided Diagnosis of Coronary Artery, Version 1.8, Shukun technology, Beijing, China). Briefly, this platform was used for evaluation of CCTA data with CAD-RADS classification which had been validated in our previous study [20]. The standardized CAD-RADS 1.0 classification was defined as the highest degree of coronary stenosis in CCTA images: CAD-RADS 0 (0%), CAD-RADS 1 (1–24%), CAD-RADS 2 (25–49%), CAD-RADS 3 (50–69%), CAD-RADS 4A (70–99% in 1 or 2 vessels), CAD-RADS 4B (left main >50% or 70–99% in 3-vessel disease), CAD-RADS 5 (total occlusion). Furthermore, the CAD-RADS 2.0 classification recommended evaluation of the total amount of coronary plaque. Because the segment involvement score (SIS) classification has been demonstrated to be more reproducible with prior evidence and can be automatically calculated in the platform, we used SIS classification to evaluate the overall plaque burden. Pearson correlation was assessed in 100 randomly selected CCTA datasets with a correlation coefficient of 0.993 between AI based-SIS and reader based-SIS (Supplementary Fig. 1). The SIS classification was based on the coronary artery plaque detection. First, curve plannar reformat (CPR), multiple planner reformat (MPR), maximum intensity projection (MIP) and volume rendering (VR) images were reconstructed based on coronary tree segmentation. Then a modified 3D V-Net architecture was trained to construct a plaque recognition model. Two probability curves along the straightened vessel images were provided by the output layer of this model, corresponding to non-calcified and calcified plaques respectively. The locations of non-calcified plaques overlap refers to the mixed plaque. The SIS can be calculated by assigning a score of 1 for each of the 18 coronary segments with any detectable plaque [20,23] (Supplementary Fig. 2). SIS 0 is defined as absence of plaque in the present study, though this classification is not recommended as a classification in the updated version of CAD-RADS. The remaining SIS classification was as follows: SIS classification 1 (score 1–2), SIS classification 2 (score 3–4), SIS classification 3 (score 5–7) and SIS classification 4 (score ≥8). In addition, The Computed Tomography Fractional Flow Reserve (CT-FFR) values were non-invasively commutated through a deep CNN algorithm in this platform using CCTA images. This platform of CT-FFR in the present study has been approved by National Medical Products Administration (NMPA No. 20233210146, https://www.nmpa.gov.cn/). The machine learning-derived CT-FFR consists of two main components, the coronary arteries segmentation model and the computational fluid dynamics (CFD) simulation model. First, 3-dimensional (3D) U-Net architecture added a Bottle-Neck design for segmentation coronary arteries and aorta, then a Growing Iterative Prediction Network (GIPN) model was developed to solve the problem of vascular segmentation fracture, final the full coronary tree segmentation was obtained [22]. The final reduced-order CFD model is applied to compute the flow and pressure of blood and calculate CT-FFR values automatically for all points along coronary arteries [24] (Supplementary Fig. 3). The CT-FFR classification was divided into three groups: CT-FFR >0.8 (positive result), CT-FFR 0.76–0.80 (borderline) and CT-FFR ≤0.75 (negative result) according to previous recommendations [7,25]. Finally, CAD-RADS 2.0 was the combination of CAD-RADS classification (stenosis severity), SIS classification (plaque burden) and CT-FFR classification (ischemia).

### 2.4 Follow-up

The follow-up procedures were approved by the institutional review boards of our hospital. We defined the primary endpoint events as MACE inclusive of all-cause mortality or MI. MACE status was determined by querying the local Community Health Service Centers. For MACE which occurred outside the city, we reviewed the medical records or contacted patients by telephone to confirm the outcome. The follow-up was done by two experts of centers for disease control and prevention (CDC) and these was blinded to other information of the patient. The deadline date of follow-up was September 30, 2022.

### 2.5 Statistical analysis

Continuous variables are shown as mean (±SD) and categorical variables are expressed as frequencies and percentages. Student's t-test was used to compare continuous variables between groups and chi-square test was used for the comparison of categorical variables. Cumulative event-free survival was estimated by the Kaplan-Meier method and the log-rank test was used for comparison between groups. Hazard ratio (*HR*) with 95% confidence intervals (*95% CI*) was calculated by univariate and multivariate Cox proportional hazard analysis. Multivariate Cox analysis was adjusted by gender, age, smoking status, alcohol consumption, hypertension, diabetes and triglyceride levels. The discriminatory values of CAD-RADS classification, SIS grade and CT-FFR classification for MACE were performed by c-statistic. We combined CAD-RADS classification 4 and 5 as one composite degree (classification 4&5) because these classifications had a low individual prevalence. *P* < 0.05 was considered to be statistically significant. All statistical analyses were carried out using R statistical package (version 4.0, R foundation for Statistical Computing, Vienna, Austria), SPSS (version 18, SPSS, Inc., Chicago, IL, USA) and MedCalc Statistical Software (version16.8.4 Ostend, Belgium).

## 3 Results

Finally, of the 2131 consecutive patients who underwent CCTA, 1796 patients (84.3%) were included for analysis in the current study. Exclusion criteria were prior MI or coronary revascularization, missing data, non-diagnostic imaging or loss to follow-up. Of the 1796 patients included in the present study, the mean age was 60.9 ± 10.4, and 44.6% (801 of 1796) were men, the basic characteristics of all patients included in the analysis are presented in Table 1.

**Table 1:** Basic characteristics of the patients included in the analysis (N = 1796).

Variables		Values
Age (years)		60.9 ± 10.4
Gender (male, n, %)		801 (44.60%)
Smoke		401 (22.33%)
Drink		226 (12.58%)
Hypertension		792 (44.10%)
Diabetes		374 (20.82%)
Triglyceride (mmol/L)		1.79 ± 1.58
Total Cholesterol (mmol/L)		4.97 ± 1.12
High density lipoprotein-C (mmol/L)		1.33 ± 0.33
Low density lipoprotein-C (mmol/L)		3.09 ± 0.96
Blood urea nitrogen (mmol/L)		5.24 ± 1.65
Creatinine (μmol/L)		64.91 ± 22.63
CAD-RADS		
0		781 (43.49%)
1		119 (6.63%)
2		548 (30.51%)
3		230 (12.81%)
4&5		118 (6.57%)
SIS		
0		751 (41.82%)
1		526 (29.29%)
2		246 (13.70%)
3		219 (12.19%)
4		54 (3.01%)
CT-FFR		
>0.8		1386 (77.17%)
0.76–0.80		196 (10.91%)
≤0.75		214 (11.92%)

CAD-RADS = Coronary Artery Disease Reporting and Data System, SIS = Segment involvement score, CT-FFR = Computed Tomography Fractional Flow Reserve.

### 3.1 Incidence of MACE

**Fig. 2:**
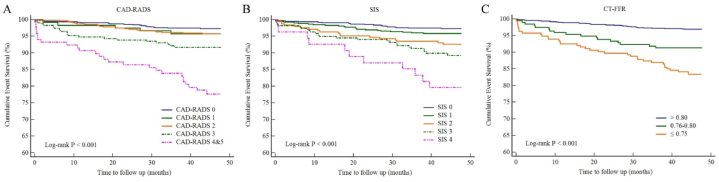
Cumulative event survival of follow-up by CAD-RADS, SIS and CT-FFR. A, Kaplan-Meier Curves of CAD-RADS; B, Kaplan-Meier Curves of SIS; C, Kaplan-Meier Curves of CT-FFR. CAD-RADS = Coronary Artery Disease Reporting and Data System, SIS = Segment involvement score, CT-FFR = Computed Tomography Fractional Flow Reserve.

In total, 94 (5.2%) MACE occurred over the median follow-up of 45.25 months (interquartile range 43.53–46.63 months). The annualized MACE rate was 0.014 (*95% CI*: 0.011–0.017). In CAD-RADS classification, the annualized MACE rate was 0.007 (*95% CI*: 0.005–0.011), 0.011 (*95% CI*: 0.005–0.025), 0.011 (*95% CI*: 0.007–0.016), 0.022 (*95% CI*: 0.014–0.033), and 0.058 (*95% CI*: 0.041–0.080) for CAD-RADS 0, CAD-RADS 1, CAD-RADS 2, CAD-RADS 3, and CAD-RADS 4&5, respectively. Similarly, the annualized MACE rate gradually increased with the increase in the SIS grade, which gave an annualized MACE rate of 0.007 (*95% CI*: 0.005–0.011), 0.011 (*95% CI*: 0.007–0.017), 0.019 (*95% CI*: 0.012–0.030), 0.028 (*95% CI*: 0.019–0.040), and 0.054 (*95% CI*: 0.031–0.087) for SIS 0, SIS 1, SIS 2, SIS 3 and SIS 4, respectively. In addition, the annualized MACE rate was 0.008 (0.006–0.011), 0.023 (0.015–0.036), and 0.043 (0.032-0.058) for CT-FFR >0.8, CT-FFR between 0.76 and 0.80 and CT-FFR ≤0.75, respectively (Table 2). Kaplan-Meier survival curves indicated that the CAD-RADS classification, SIS grade, and CT-FFR classification were all significantly associated with the increase in the cumulative MACE (all *P* < 0.001) (Fig. 2A–C).

**Table 2:** Incidence of MACE.

	No. of patients		No. of MACE (%)		Annualized MACE rate	
Overall	1796		94 (5.2)		0.014 (0.011–0.017)	
CAD-RADS						
0	781		21 (2.7)		0.007 (0.005–0.011)	
1	119		5 (4.2)		0.011 (0.005–0.025)	
2	548		23 (4.2)		0.011 (0.007–0.016)	
3	230		19 (8.3)		0.022 (0.014–0.033)	
4&5	118		26 (22.0)		0.058 (0.041–0.080)	
SIS						
0		751		20 (2.7)		0.007 (0.005–0.011)
1		526		22 (4.2)		0.011 (0.007–0.017)
2		246		18 (7.3)		0.019 (0.012–0.030)
3		219		23 (10.5)		0.028 (0.019–0.040)
4		54		11 (20.4)		0.054 (0.031–0.087)
CT-FFR						
>0.8		1386		42 (3.0)		0.008 (0.006–0.011)
0.76–0.80		196		17 (8.7)		0.023 (0.015–0.036)
≤0.75		214		35 (16.3)		0.043 (0.032–0.058)

CAD-RADS = Coronary Artery Disease Reporting and Data System, SIS = Segment involvement score, CT-FFR = Computed Tomography Fractional Flow Reserve, MACE = Major adverse cardiovascular events.

### 3.2 Univariable and multivariable cox

CAD-RADS classification, SIS grade, and CT-FFR classification were all significantly associated with the endpoint in univariate and multivariate Cox analysis. The risk for MACE increased from *HR* 1.07 (*95% CI*: 0.36–3.18) for CAD-RADS 1 to 4.13 (*95% CI*: 2.07–8.25) for CAD-RADS 4 and 5 in multivariate Cox analysis. The SIS grade was significantly associated with a higher risk of MACE in univariate (*HR* 1.59, *95% CI*: 0.87–2.91 for SIS 1 to *HR* 8.35, *95% CI*: 4.00–17.42 for SIS 4) and multivariate cox analysis (*HR* 0.99, *95% CI*: 0.50–1.97 for SIS 1 to *HR* 2.78, *95% CI*: 1.06–7.27 for SIS 4). The *HR* of the risk of MACE for CT-FFR between 0.76 and 0.80, CT-FFR ≤0.75 was 2.12 (*95% CI*: 1.14–3.93) and 3.65 (*95% CI*: 2.17–6.14) in multivariate Cox analysis respectively, using CT-FFR >0.8 as the reference group (Table 3).

**Table 3:** Univariable and multivariable cox.

	Unadjusted		Adjusted	
	HR (95% CI)	P Value	HR (95% CI)	P Value
CAD-RADS				
0	Reference		Reference	
1	1.58 (0.60–4.18)	0.360	1.07 (0.36–3.18)	0.899
2	1.58 (0.87–2.85)	0.132	1.02 (0.53–1.98)	0.954
3	3.19 (1.82–5.93)	<0.001	2.00 (1.00–3.98)	0.049
4&5	9.08 (5.11–16.15)	<0.001	4.13 (2.07–8.25)	<0.001
SIS				
0	Reference		Reference	
1	1.59 (0.87–2.91)	0.134	0.99 (0.50–1.97)	0.981
2	2.83 (1.50–5.35)	0.001	1.72 (0.85–3.48)	0.131
3	4.11 (2.25–7.47)	<0.001	2.44 (1.24–4.80)	0.01
4	8.35 (4.00–17.42)	<0.001	2.78 (1.06–7.27)	0.04
CT-FFR				
>0.8	Reference		Reference	
0.76–0.80	2.97 (1.69–5.21)	<0.001	2.12 (1.14–3.93)	0.017
≤0.75	5.79 (3.70–9.07)	<0.001	3.65 (2.17–6.14)	<0.001

CAD-RADS = Coronary Artery Disease Reporting and Data System, SIS = Segment involvement score, CT-FFR = Computed Tomography Fractional Flow Reserve, HR = Hazard ratio, CI = Confidence intervals.

### 3.3 Comparison of the prediction value of MACE between CAD-RADS 1.0 and CAD-RADS 2.0

**Fig. 3:**
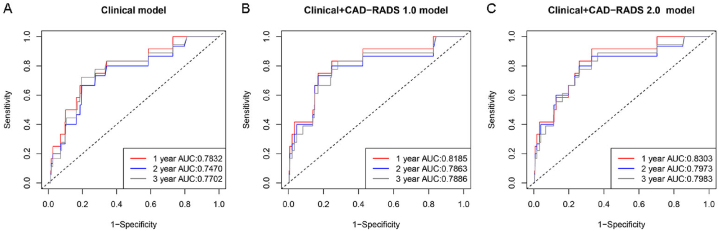
Time dependent ROC curves for MCAE. A, Clinical model; B, Clinical + CAD-RADS 1.0 model; C, Clinical + CAD-RADS 2.0 model.

The capability of predicting further MACE for the CAD-RADS 1.0, SIS grade and CT-FFR classification were c-statistic 0.681 (*95% CI*: 0.624–0.738), c-statistic 0.670 (*95% CI*: 0.615–0.725) and c-statistic 0.676 (*95% CI*: 0.623–0.729), respectively. CAD-RADS 1.0, SIS grade and CT-FFR classification showed similar discriminatory values compared with each other (all *P* > 0.05). Data from CCTA using CAD-RADS 2.0 (CAD-RADS 1.0 + SIS grade + CT-FFR classification) showed further incremental increases in the prognostic value to predict MACE (c-statistic 0.702, *95% CI*: 0.641–0.763, *P* = 0.047), compared with CAD-RADS 1.0 (Table 4). The prognostic performances of all three models are shown in Fig. 3 A-C. The Clinical + CAD-RADS 2.0 model showed the highest values of the area under the time dependent receiver operating characteristic (ROC) curve.

**Table 4:** Incremental Value of CAD-RADS 2.0 in the overall population.

Univariable Model	C-Statistic	P Value	Multivariable Models	C-Statistic	P Value
CAD-RADS 1.0	0.681 (0.624–0.738)	0.305^#^			
SIS	0.670 (0.615–0.725)	0.674^$^			
CT-FFR	0.676 (0.623–0.729)	0.769^^^	CAD-RADS 2.0	0.702 (0.641–0.763)	0.047*

CAD-RADS = Coronary Artery Disease Reporting and Data System, SIS = Segment involvement score, CT-FFR = Computed Tomography Fractional Flow Reserve.

^#^ CAD-RADS 1.0 vs. SIS; ^$^ SIS vs. CT-FFR; ^^^ CAD-RADS 1.0 vs. CT-FFR; * CAD-RADS 1.0 vs. CAD-RADS 2.0.

## 4 Discussion

The present study found that CAD-RADS 1.0, SIS grade and CT-FFR classification were all significantly associated with MACE in patients with suspected CAD during the median follow-up of 45 months. These classifications showed similar discriminatory value compared with each other. Furthermore, the combined classification (CAD-RADS 2.0) had greater prognostic value for further MACE than CAD-RADS 1.0. Together, these findings support the prognostic value of MACE using CAD-RADS 2.0 for standardized reporting of CCTA. To the best of our knowledge, this study is the first to investigate the prognostic value of CAD-RADS 2.0 compared with CAD-RADS 1.0.

Structured and standardized reporting of CCTA remains the primary goal of CAD-RADS. High diagnostic accuracy (96.8%–100%) and high reproducibility (ICC = 0.9862) was reported to be better correlated with grade of stenosis compared to invasive coronary angiography in a recent study [26]. Moreover, Muscogiuri et al. have developed a deep CNN for automatically classifying patients using CAD-RADS and achieved an accuracy of 60%–86% [27]. We have previously developed a standardized reporting of deep CNN based CAD-RADS using CCTA images, which can accurately and rapidly evaluate patients with suspected CAD [20]. In addition to these widely reported diagnostic values, the CAD-RADS classification has been demonstrated to have high prognostic value for MACEs in the real word [[9], [10], [11]]. However, few studies have focused on evaluating the prognosis of CAD-RADS classification using deep learning methods. The deep CNN-based CAD-RADS classification showed a moderate prognostic value for predicting MACE in patients with suspected CAD.

The prognostic value of CAD-RADS has been well established in multicenter studies recently. However, the comparison of prognostic value between the CAD-RADS classification and the traditional CAD classifications is still controversial. Bittner et al. found that CAD-RADS classification based on CCTA had greater predictive value for MACE than traditional CAD classifications in the Prospective Multicenter Imaging Study for Evaluation of Chest Pain (PROMISE) trial with 3840 eligible patients [11]. In contrast, recent large sample studies revealed that the prognostic value of CAD-RADS classification for predicting the risk of MI or all-cause death was non-inferior to traditional CAD classifications [13,14]. Moreover, the plaque burden has been regarded as a high risk factor for further MACE or death and is therefore recommended to be evaluated, especially in individuals without obstructive CAD, as accumulated studies have demonstrated that most events occur in patients without obstructive CAD [14,28,29]. The present study showed similar results to previous studies. Only a few studies have investigated the prognostic value between CAD-RADS and plaque burden. A recent prospective multicenter study of 3840 patients with stable chest pain and suspected CAD found that CAD-RADS had significantly higher discriminatory value for predicting cardiovascular events than CAC (C-statistic, 0.747 vs. 0.657, *P* < 0.001) [11]. On the other hand, the results from the recent Western Denmark Heart Registry (WDHR) study showed that plaque burden was the main predictor of risk for further MACE and death compared to the traditional CAD classification in 23759 symptomatic patients who underwent CCTA [16]. The present study showed that there was no significant difference in discriminatory value for predicting further cardiovascular events between CAD-RADS and plaque burden. Which classification has a better prognostic value is still unclear according to the current evidence. However, the consistent conclusion is that the combined model (CAD-RADS classification plus plaque burden) had higher risk prediction benefits than any single classification model [10,11]. The current study showed similar results to these findings.

Anatomic stenosis or plaque burden on CCTA have been reported to result in poor prediction in the hemodynamic significance of lesions [30]. The recent Fractional Flow Reserve Versus Angiography in Multivessel Evaluation 2 (FAME 2 trial) showed that functional assessment (FFR) was more important for predicting the natural history of stenoses than anatomy evaluation in patients with suspected CAD [31]. Some studies showed that CT-FFR had superior outcome predictive value than anatomic stenosis based on CCTA [18,19,32]. However, the anatomic stenosis classification was merely classified into two groups (<50%, ≥50% groups or < 70%, ≥70% groups) based on CCTA images. Moreover, the comparison of prognosis between the CAD-RADS classification and CT-FFR classification has only been reported in few studies [[33], [34], [35]]. In these studies, CT-FFR classification was divided into two groups (CT-FFR >0.8 and CT-FFR ≤0.8) as high agreement was found using the threshold of 0.8 between CT-FFR and invasive FFR. However, the correlation between CT-FFR and invasive FFR has recently been reported to be uncertain in the borderline CT-FFR values between 0.76 and 0.8 [36,37]. The present study further demonstrated that patients with CT-FFR ranging between 0.76 and 0.8 had a better prognosis than those with CT-FFR ≤0.75 as shown by Kaplan-Meier analysis. Therefore, these patients may benefit from further re-stratification. Admittedly, no significant difference was found in the discriminatory value for predicting further cardiovascular events between CT-FFR and CAD-RADS. Together, the present data showed that CAD-RADS 2.0 had a higher discriminatory predictive value than CAD-RADS 1.0, SIS or CT-FFR classification, which is not involved in previous studies.

Despite the important findings and clinical implications of prognostic value for CAD-RADS 2.0 in patients with suspected CAD, this study had several limitations. First, the study contains a relatively small sample size and selection bias may be present due to the retrospective nature of this study, larger samples and multicenter studies are needed to reduce bias. Second, we excluded CAD-RADS modifiers (stents, S; vulnerable plaque features, V and grafts, G) as these did not prove the accuracy of CNN-based CCTA in the current version and they were not applicable to the evaluation of CT-FFR according to the current guidelines. Third, the present study conflated CAD-RADS classifications 4 and 5 because of the low prevalence. Fourth, due to the unavailability of the data on specific causes of death, the clinical endpoint was defined as all-cause mortality or MI. Cardiac mortality could not be separately assessed as an additional outcome which would be expected to have a stronger correlation with atherosclerotic burden. Lastly, only SIS was used to assess plaque burden, while other methods may result in different accuracies, further studies are needed to investigate the difference.

In conclusion, the novel CAD-RADS 2.0 evaluated by CNN-based CCTA showed higher prognostic value of MACE than CAD-RADS 1.0 in patients with suspected CAD.

## Financial disclosure

This work was supported by the Scientific Research Project of Wuhan Municipal Health Commission (WX2019B02, WX20D49) and Health Commission of Hubei Province scientific research project (WJ2019H425). The funders had no role in study design, data collection and analysis, decision to publish, or preparation of the manuscript.

## Author contribution statement

Xiang Wang and Yun Hu conceived and designed the experiments.

All the authors performed the experiments.

Zengfa Huang and Yang Yang analyzed and interpreted the data.

Beibei Cao, Mei Li, Xinyu Du, Xi Wang, Zuoqin Li, Wanpeng Wang, Yi Ding and Jianwei Xiao contributed reagents, materials, analysis tools or data.

Zengfa Huang, Yang Yang, Zheng Wang, Yunting Hu and Yun Hu wrote the paper.

## Data availability statement

Data will be made available on request.

## Author contributions statement

Xiang Wang and Yun Hu conceived and designed the experiments, All the authors performed the experiments, Zengfa Huang and Yang Yang analyzed and interpreted the dat, Beibei Cao, Mei Li, Xinyu Du, Xi Wang, Zuoqin Li, Wanpeng Wang, Yi Ding and, Jianwei Xiao contributed reagents, materials, analysis tools or data, Zengfa Huang, Yang Yang, Zheng Wang, Yunting Hu and Yun Hu wrote the paper.

## Declaration of competing interest

The authors declare they have no competing interests.
