# Supplementary material for: Comparison of prognostic value between CAD-RADS 1.0 and CAD-RADS 2.0 evaluated by convolutional neural networks based CCTA
Source: Heliyon. 2023 May 4;9(5):e15988. doi: 10.1016/j.heliyon.2023.e15988 (PMC10195897; doi:10.1016/j.heliyon.2023.e15988)

**Supplementary Figure 1** Correlation between AI-based SIS and reader-based SIS.

**Supplementary Figure 2** Deep learning (DL)-based fully Automatic plaque detection.

**Supplementary Figure 3** Display the platform of CT-FFR.

**Supplementary Figure 1**

**
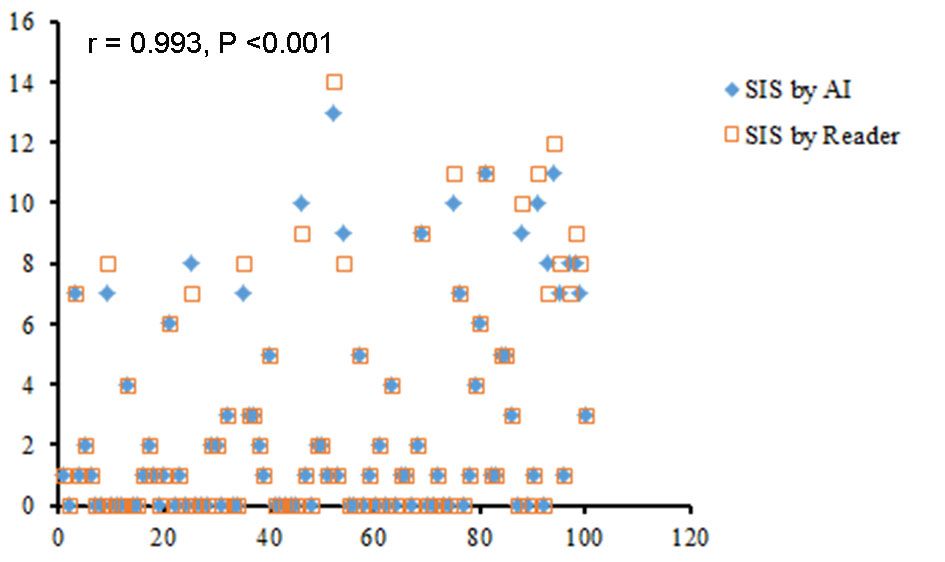
**

**Supplementary Figure 2**

**
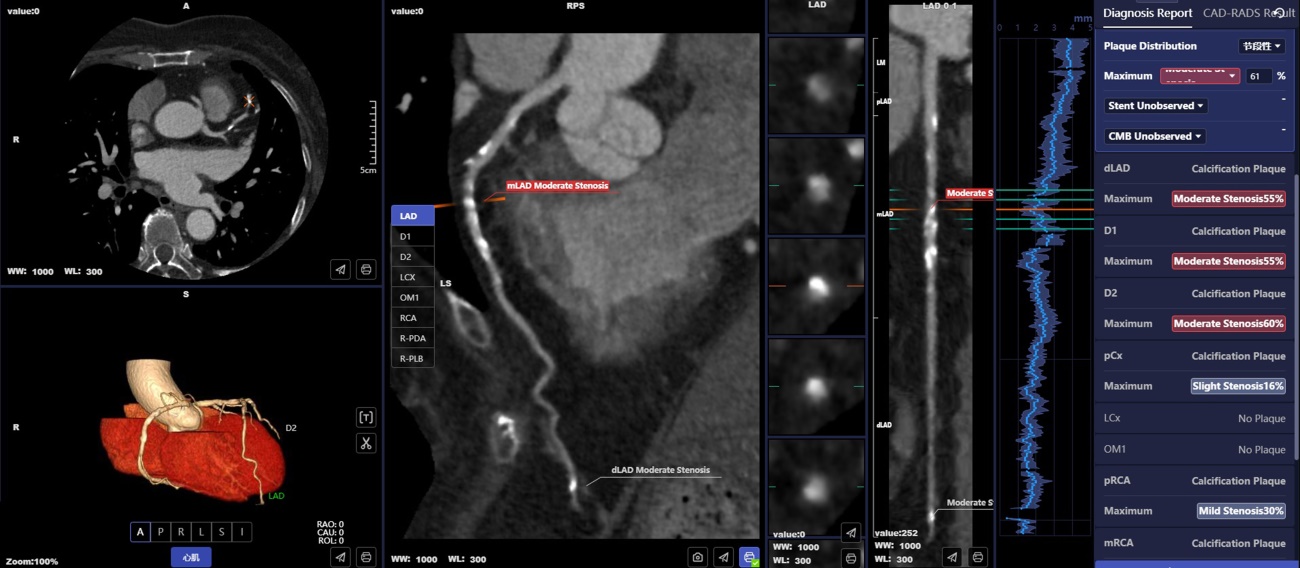
**

**Supplementary Figure 3**


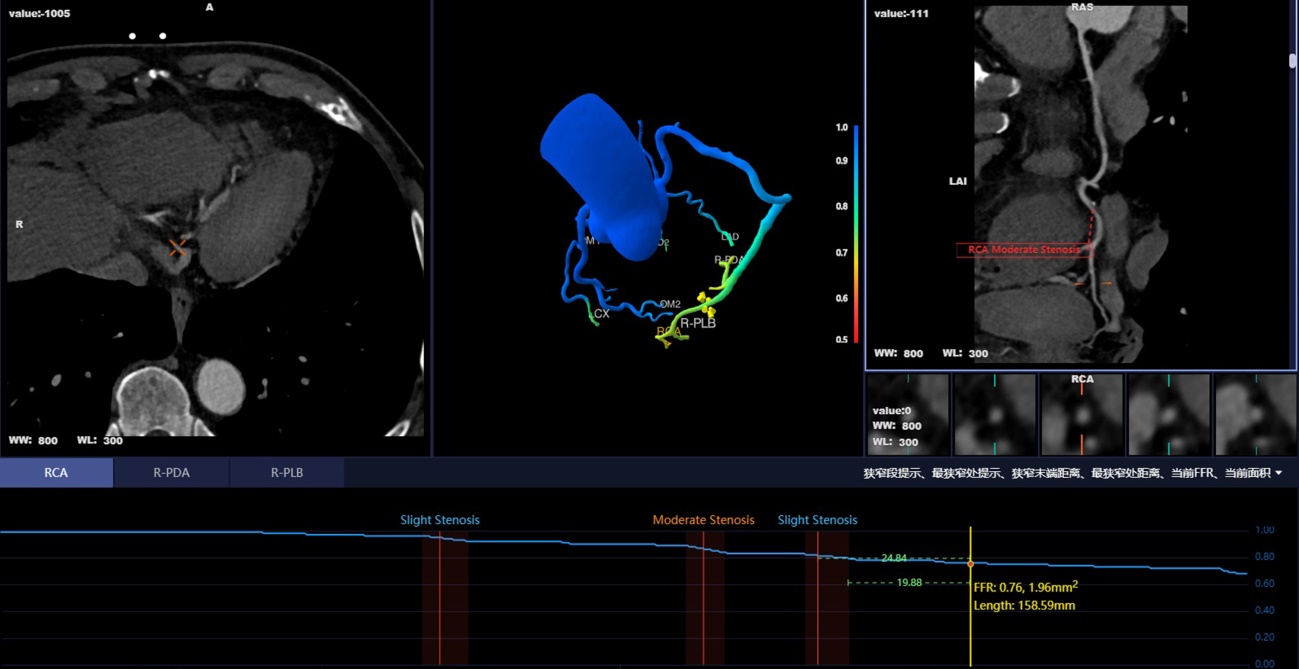

Supplement: Multimedia component 1 [file mmc1.docx]
